# Good vibrations, bad vibrations: Oscillatory brain activity in the attentional blink

**DOI:** 10.2478/v10053-008-0089-x

**Published:** 2011-12-22

**Authors:** Jolanda Janson, Cornelia Kranczioch

**Affiliations:** 1 Neuropsychology Lab, Department of Psychology, Carl von Ossietzky University, Oldenburg, Germany; 2 Department of Neurology, Biomagnetic Center, University Hospital Jena, Jena, Germany

**Keywords:** oscillatory brain activity, attentional blink, EEG, review, visual attention

## Abstract

*The attentional blink* (AB) is a deficit in reporting the second (T2) of two targets (T1, T2) when presented in close temporal succession and within a stream of distractor stimuli. The AB has received a great deal of attention in the past two decades because it allows to study the mechanisms that influence the rate and depth of information processing in various setups and therefore provides an elegant way to study correlates of conscious perception in supra-threshold stimuli. Recently evidence has accumulated suggesting that oscillatory signals play a significant role in temporally coordinating information between brain areas. This review focuses on studies looking into oscillatory brain activity in the AB. The results of these studies indicate that the AB is related to modulations in oscillatory brain activity in the theta, alpha, beta, and gamma frequency bands. These modulations are sometimes restricted to a circumscribed brain area but more frequently include several brain regions. They occur before targets are presented as well as after the presentation of the targets. We will argue that the complexity of the findings supports the idea that the AB is not the result of a processing impairment in one particular process or brain area, but the consequence of a dynamic interplay between several processes and/or parts of a neural network.

## Introduction

**Figure 1.**
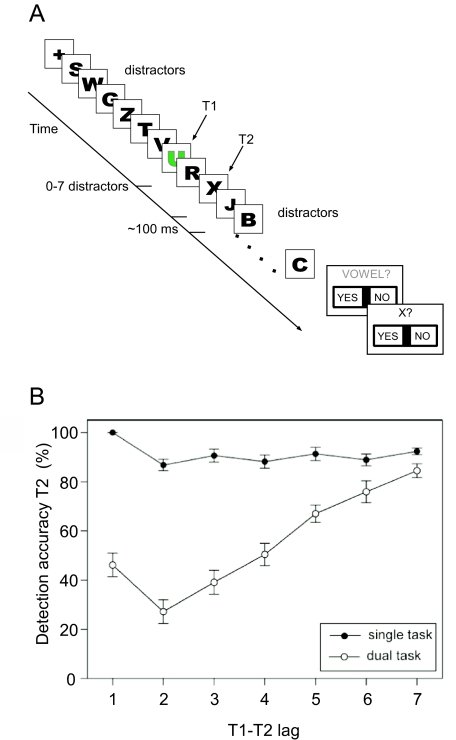
(a) Stimuli and trial structure typically used in an attentional blink (AB) paradigm. A stream of distractor stimuli of variable length is presented before the first target (T1). After the first target (T1) and the second target (T2) more distractors are shown. A trial finishes with non-speeded responses regarding the targets. All stimuli are normally presented at a rate of about 10 per second, resulting in rapid serial visual presentation (RSVP). The critical condition is the dual target task where both targets are task relevant. Typical control conditions include the case where both targets are shown but only T2 is task relevant (single task condition), where the RSVP contains only T1, or where no targets are contained in the RSVRSVRSVP. These conditions help to differentiate between distractor- and target-related brain activity. (b) Illustration of the behavioural AB effect. The graph shows the mean detection accuracy for the second target (T2) as a function of lag between T1 and T2. A T1-T2 lag of 1 indicates that T2 was the first stimulus after T1, etc. Note that in the single task condition − when T1 can be ignored −T2 performance is very high. The AB is observed when both targets are task relevant, that is, in the dual task condition. T2 performance is particularly impaired for intermediate T1-T2 lags, which corresponds to a time window of about 200 to 500 ms after T1 presentation. The relatively better performance for the shortest T1-T2 lag has become known as T1-sparing. Adapted from “Event-Related Potential Correlates of the Attentional Blink Phenomenon” by C. Kranczioch, S. Debener, and A. Engel, 2003, *Brain Research. Cognitive Brain Research, 17*(1), pp. 179, 181.

Attention is distributed in time: We are quicker to respond to an event that happens at the moment in time we expect it or that is in the focus of temporal attention (Coull, 2004). And yet our ability to voluntarily distribute attentional resources in time is limited. When two targets need to be identified amongst a rapid stream of distractor stimuli (see Figure 1a) a deficit for identifying the second target is evident. The deficit disappears if only the second target needs to be identified (see Figure 1b). This so-called *attentional blink* (AB) is a transitory attention impairment that is most pronounced when the second target (T2) is presented 200-500 ms after the first target (T1). It was first reported in 1987 (Broadbent & Broadbent, 1987; Weichselgartner & Sperling, 1987) and received its name 5 years later from Raymond and colleagues (Raymond, Shapiro, & Arnell, 1992) who where the first to study it in greater detail.

 Since the discovery of the AB it has been the topic of a vast amount of experiments, among others because it allows to study neural correlates of conscious visual awareness by “rendering the visible invisible” (Kim & Blake, 2005, p. 381). In numerous behavioural studies, properties of the distractor stream as well as the targets have been manipulated. This research has led to an increasing number of theories on the origins of the AB; research and theories have been extensively reviewed in recent reviews (Dux & Marois, 2009; Martens & Wyble, 2010). One of the earliest models of the origins of the AB blink is the two-stage model proposed by Chun and Potter (1995) and its adaptation by Potter, Staub, and O’Connor (2002) . It states that in Stage 1 stimuli activate stored conceptual representations but in order to avoid “overwriting” by subsequently presented stimuli each stimulus has to be encoded and consolidated in working memory. This second processing stage has, however, a limited processing capacity and as a consequence, stimuli have to compete for access to it. When T2 follows T1 in close temporal proximity it has to wait to gain access to Stage 2, which makes it vulnerable to decay and overwriting. Thus the AB is seen as a consequence of a bottleneck in working memory consolidation. Another early model assuming some kind of central capacity limitation is the interference theory by Shapiro, Raymond, and Arnell (1994) . This theory suggests that T1 and T2, but also the T1+1 and the T2+1 stimuli enter working memory. All are assigned a weighting that depends on the space available in the store and their similarity to target templates. It is assumed that T2 is prone to fail to be retrieved from working memory because it receives a diminished weighting and is more open to interference from other items in the store. 

More recently theories have shifted from assuming central capacity limitations as underlying the AB towards the assumption of a critical role of the configuration of the attention network. For example, in the delayed attentional reengagement account by Nieuwenstein and colleagues (Nieuwenstein, 2006; Nieuwenstein, Chun, van der Lubbe, & Hooge, 2005; Nieuwenstein & Potter, 2006; Nieuwenstein, Potter, & Theeuwes, 2009) it has been proposed that the AB is the result of the dynamics of attentional selection: A top-down process that makes sure that attention is engaged to T1 and disengaged as soon as T1 disappears cannot react fast enough to re-engage to T2. The overinvestment theory (Olivers & Nieuwenhuis, 2006; Shapiro, Schmitz, Martens, Hommel, & Schnitzler, 2006) is another example of a model that puts great emphasis on attentional factors. Similar to central capacity limit models it is assumed that neural representations of targets and distractors interfere and compete for access to a capacity limited stage. However, the processing interference in the capacity limited processing stage is a direct consequence of allocating too many attentional resources to the distractor stream and/or T1, hence there are not enough resources left for processing both T1 and T2.

 Soon after the seminal work by Raymond et al. (1992) , researchers began to study the AB by means of electro-encephalographic (EEG) measurements in order to better understand which aspects of target processing are modulated in the AB. In 1996 the first event-related potential (ERP) study on the AB was published by Luck and colleagues (Luck, Vogel, & Shapiro, 1996). They used the N400 ERP to demonstrate that even though an AB is seen in the behavior, T2 items that remain undetected are nevertheless processed to the point of meaning extraction, that is, a late stage in the processing pathway. In a later publication, the same research group could show that in spite of the unimpaired N400, the P3 ERP component, which is suggested to reflect updating of working memory (Luck, 2005), was absent for undetected T2’s within the AB time-window. No differences were found for the N1 and P1 ERP components which signify sensory processing (Vogel, Luck, & Shapiro, 1998). Since then a number of studies have replicated, refined, and supplemented these early findings (e.g., Dell’Acqua, Sessa, Jolicoeur, & Robitaille, 2006; Jolicoeur, Sessa, Dell’Acqua, & Robitaille, 2006; Kranczioch, Debener, & Engel, 2003; Kranczioch, Debener, Maye, & Engel, 2007; Luck et al., 1996; Martens, Munneke, Smid, & Johnson, 2006; Rolke, Heil, Streb, & Hennighausen, 2001; Sergent, Baillet, & Dehaene, 2005; Verleger et al., 2009, 2010). For instance, based on studies that compared ERPs in trials in which T2 was detected and in which it was missed (Kranczioch et al., 2003; Rolke et al., 2001), it is now commonly agreed that the P3 to T2 is not generally suppressed in the AB, but only if T2 is not detected. Also, evidence is accumulating that suggests that the modulation of the P3 component is not the earliest signature of the AB deficit but that differences are already evident in the T2-related N2 (Kranczioch et al., 2007; Sergent et al., 2005) and N2pc components (Dell’Acqua et al., 2006; Jolicoeur et al., 2006), likely to reflect processes related to attentional selection. Taken together, the picture that emerges from ERP research suggests that the AB occurs after perceptual and conceptual representations have been formed, that is, at a relatively late stage of processing,

 One of the great strengths of ERPs is without doubt their high temporal resolution, which allows following information processing on a millisecond scale. However, ERPs lack in spatial resolution. This is the strength of functional magnetic resonance imaging (fMRI) which allows identifying brain regions associated with a particular task or phenomenon with high spatial precision. Early fMRI-studies that aimed at identifying the brain areas critically involved in the AB chose experimental manipulations that included only one target (Marois, Chun, & Gore, 2000) or compared the summed activation of T1 and T2 (Feinstein, Stein, Castillo, & Paulus, 2004; Kranczioch, Debener, Schwarzbach, Goebel, & Engel, 2005; Marcantoni, Lepage, Beaudoin, Bourgouin, & Richer, 2003). In the study conducted by Marois et al. (2000) , neural correlates of the AB were studied by comparing several conditions that included only T1 but varied in the degree of interference of the distractors. In separate behavioral experiments the degree of interference of the distractors had been shown to modulate T2 performance, and thus the AB. The fMRI results indicated that highly interfering distracters that increased the AB were associated with higher activation in the right intra-parietal and frontal cortex as compared to the low interference conditions (Marois et al., 2000). Marcantoni et al. (2003) compared the summed neural activation for T1 and T2 in a condition where T2 was presented within the AB window (lag 3) and in a condition where T2 was presented outside the AB window (lag 7). They found increased activation in the cerebellum, the frontal, inferotemporal, and posterior parietal cortex in the lag 3 compared to the lag 7 condition. Feinstein et al. (2004) compared “blinkers”, people who in an AB paradigm often have an AB, with “non-blinkers”, people who rarely have an AB. They found that non-blinkers on average show an increase in activation in the anterior cingulate, the medial prefrontal cortex, and the right superior frontal gyrus during the AB-task when compared to the blinkers that peaked earlier than the estimated peak of the hemodynamic response. The blinkers, in contrast, showed a decrease in activation in these areas compared to the estimated hemodynamic response function. Finally, Kranczioch et al. (2005) compared AB with no-AB trials collapsed over lag 1 and lag 2 conditions and found more activation in the right lateral occipital complex and the bilateral fusiform gyrus in AB compared to no-AB trials. This activation preceded activation in the frontal and parietal areas, which was higher in the no-AB trials compared to the AB trials. Taken together, the results of these early studies point towards a fronto-temporo-parietal network as a possible locus of the AB deficit. 

The first study to attempt to differentiate between the neural activation for T1 and T2 visual “object” areas used face stimuli for T1 and scenes for T2 (Marois, Yi, & Chun, 2004). The parahippocampal place area (PPA) was found to be activated in AB trials, that is, even when T2 was not consciously perceived. The blood oxygenation level dependent (BOLD) signal was smaller though than in the no-AB trials, which was interpreted as reflecting that increased neural responses in object processing regions accompany awareness of T2. Results of other studies (Kranczioch et al., 2005; Shapiro, Johnston, Vogels, Zaman, & Roberts, 2007) contradict this interpretation, however, by finding more BOLD activation in object processing regions if T2 remains undetected. Johnston, Shapiro, Vogels, and Roberts (2007; see also Shapiro et al., 2007) argue that this contradiction can be explained by considering the different task parameters: If attention and perceptual information are limited (Kranczioch et al., 2005; Shapiro et al., 2007), activation in object processing areas is enhanced and reflects the effort it takes to process a stimulus for which attention is lacking. If only perceptual information is limited (Marois et al., 2004; see also Slagter, Johnstone, Beets, & Davidson, 2010) then activation in object processing areas reflects the end product of successful perception. Several recent fMRI studies have found an increase in T2-related activation in early visual areas when T2 is detected (no-AB trials) and a decrease if T2 is missed (AB trials; Hein, Alink, Kleinschmidt, & Müller, 2009; Stein, Vallines, & Schneider, 2008; Williams, Visser, Cunnington, & Mattingley, 2008). The apparent disagreement of these results to the findings on early ERP components (Vogel et al., 1998) can be resolved if one assumes that they reflect the establishment of iterative feedback loops between higher and lower cortical areas (Di Lollo, Enns, & Rensink, 2000; Stein et al., 2008; Williams et al., 2008). In sum, the fMRI evidence so far implies the involvement of a fronto-temporal-parietal network in the AB. These higher cortical areas likely modulate activity in lower visual areas via iterative feedback loops.

As outlined above, ERP and fMRI findings generally support the idea that the AB occurs at a relatively late stage of processing. The fMRI findings suggest the involvement of a network distributed across temporal, parietal, and frontal areas that, via iterative feedback, modulates activity in lower visual areas. Yet ERPs and fMRI provide only a limited view of event-related brain-dynamics (Makeig, Debener, Onton, & Delorme, 2004). In particular, evidence from EEG and magneto-encephalogram (MEG) studies has accumulated suggesting that oscillatory signals may subserve the functions necessary to temporally coordinate the information between brain areas and thereby establish functional networks (Engel & Fries, 2010; Fries, 2005; Sauseng & Klimesch, 2008; Singer, 1999; Varela, Lachaux, Rodriguez, & Martinerie, 2001). In the following we will review the findings of modulations of oscillatory brain activity in the AB and discuss which role oscillatory brain activity may play for the occurrence of the AB.

## Some basics on oscillatory brain activity

Oscillatory brain activity can be characterized by its amplitude, its phase, and its frequency. The amplitude is defined by the amount of Microvolts (µV) that is generated, whereas the phase of an oscillation is cyclic and ranges between 0 and 2. Oscillations can be categorized into five frequency bands: delta (0-4 Hz), theta (4-8 Hz), alpha (8-12 Hz), beta (12-30 Hz), and gamma (30-80 Hz; Herrmann, Grigutsch, & Busch, 2002) though the precise frequency boundaries per band are not stringently applied and can vary from one publication to another. Oscillatory brain activity can be spontaneous or event-related (Herrmann et al., 2002). Continuous EEG can be considered to consist largely of a mix of spontaneous oscillations at different frequencies that change over time (Gutberlet, Jung, & Makeig, 2009). Event-related oscillations can further be divided into induced and evoked oscillation. Evoked oscillations are characterized by a high degree of time and phase locking to an event, whereas induced activity occurs after an event but the onset of this occurrence and its phase vary in time (Herrmann et al., 2002). AB research has so far focused on three aspects of oscillatory brain activity: amplitude, inter-trial phase consistency, and *inter-area phase locking*. The following section will give a short and general description of each of these measures and refer to the AB studies that used the respective measure.

### Amplitude

Among others, the amplitude of oscillatory brain activity is related to the number of neurons generating it. Moreover, when a large population of neurons synchronizes the phase of an ongoing oscillation, the amplitude of the oscillation will also increase (Gutberlet et al., 2009). The amplitude of an oscillation can be extracted in different ways. Among the presently most popular ones is wavelet analysis, where the frequency content of a time series is extracted by way of convolution (Herrmann et al., 2002). The advantage of wavelet analysis is that it allows observing both the frequency content of a signal and the time course of the frequency signal. This method has been used in some AB studies (Kranczioch et al., 2007; Slagter, Lutz, Greischar, Nieuwenhuis, & Davidson, 2009). Other studies restricted their analysis to only one predefined frequency, in which case a very effective way of amplitude extraction is complex demodulation (Keil & Heim, 2009; Keil, Ihssen, & Heim, 2006). In complex demodulation, a sine and cosine function at a frequency of interest (FOI) are multiplied with the data. For AB studies a natural choice for the FOI would be the presentation frequency of the stimulus sequence. The result of the multiplication is then filtered, usually with 2 or 3 Hz “space” around the FOI. After filtering, the sine and cosine time-series are combined resulting in a measure of amplitude over time for the FOI (Draganova & Popivanov, 1999; Keil & Heim, 2009; Keil et al., 2006). In the AB study by MacLean and Arnell (2011) , analysis focused on just one frequency band. Here, data were bandpass filtered and the amplitude of the filtered EEG was then squared to provide an estimate of power. 

Martens and colleagues (Martens et al., 2006; Wierda, van Rijn, Taatgen, & Martens, 2010) analyzed distractor-related activity in an AB setup. The repetitive, fast presentation of stimuli evokes oscillatory brain activity, the steady-state visual evoked potential (ssVEP; Dawson, 1954; Vialatte, Maurice, Dauwels, & Cichocki, 2010). Taking advantage of the stability of the ssVEP over trials Martens et al. compared the peak amplitude of the averaged ssVEP in different conditions, just as one would do in a standard ERP analysis.

### Inter-trial phase consistency

 Phase synchronization is seen as a central mechanism for information processing within and between brain areas (Herrmann et al., 2002). If considered for a single electrode site or brain area it provides a measure of how consistent the phase of an oscillation is from trial to trial. Phase synchronization measures the relation between the temporal structures of the signals regardless of signal amplitude. A widely used quantification is the phase-locking factor (PLF) or inter-trial phase coherence (ITC; Delorme & Makeig, 2004; Kranczioch et al., 2007; Tallon-Baudry, Bertrand, Delpuech, & Pernier, 1996) as used in the AB study of Kranczioch et al. (2007) . Phase consistency measures such as ITC or PLF range between 0 and 1, indicating for each time and frequency analysed the degree of phase consistency, with 0 indicating a random phase distribution and 1 indicating perfect phase consistency between trials. The phase-locking factor of Palva, Linkenkaer-Hansen, Näätänen, and Palva (2005) used by Slagter and colleagues (2009) is based on a similar concept. Irrespective of the quantification used in the respective studies, in the following we will refer to all these measures as inter-trial phase consistency.

### Inter-area phase locking

 The degree of phase synchronization or phase locking between a pair or group of electrodes or brain regions in sets of trials is often quantified using the phase locking index (PLI; Herrmann et al., 2002; Lachaux, Rodriguez, Martinerie, & Varela, 1999). Varela, Lachaux, Rodriguez, and Martinerie (2001) specify it as synchronization between brain areas farther apart than 1-2 cm, such as, for instance, the occipital and frontal lobes. Similar to inter-trial phase consistency, only the information about the phase of the spectral estimate of each time series is taken into account, providing a measure that is not affected by signal amplitude. Inter-area phase locking varies between 0 for no phase locking and 1 indicating perfect synchronization (Delorme & Makeig, 2004; Herrmann et al., 2002; Varela et al., 2001). Concepts similar or related to the PLI used in AB research are phase-locking value (PLV; Kranczioch et al., 2005), phase synchrony index (SI; Gross et al., 2004), and dynamic cross-lag phase synchronization (dcPSI; Nakatani, Ito, Nikolaev, Gong, & van Leeuwen, 2005), all of which will in the following be referred to as inter-area phase locking. 

It should be noted though that despite the increasing use of inter-area phase locking as a dependent measure, its interpretability is limited due to the ambiguity of relating a signal measured at the surface (EEG or MEG) to a particular brain region. Moreover, oscillatory activity in one brain region will be recorded by almost all electrodes/sensors, making the analysis susceptible to artefactual phase locking (Hoechstetter et al., 2004; Nunez et al., 1997, 1999). One possible way to solve this problem is to transform the surface signals into source space and then to analyse phase locking between sources, not electrodes or sensors (Gross et al., 2001; Hoechstetter et al., 2004). So far only one study on the AB has taken this approach (Gross et al., 2004). Another critical issue that should be kept in mind when running frequency analyses in general and calculating inter-area phase locking in particular is that the choice of reference can have massive effects on the results (Nunez et al., 1997, 1999; Trujillo, Peterson, Kaszniak, & Allen, 2005). Moreover, if the number of trials entering phase locking analysis is too small, phase locking can be over- or underestimated (Nunez et al., 1997, 1999). Results of a time-frequency analysis will also depend on how the FOI and time windows are chosen and whether the para-meters of the analysis are set such that they give optimal resolution in the time or the frequency domain (Trujillo et al., 2005).

## Oscillatory Activity in the Attentional Blink

### Models on the role of oscillatory activity in the attentional blink

**Figure 2.**
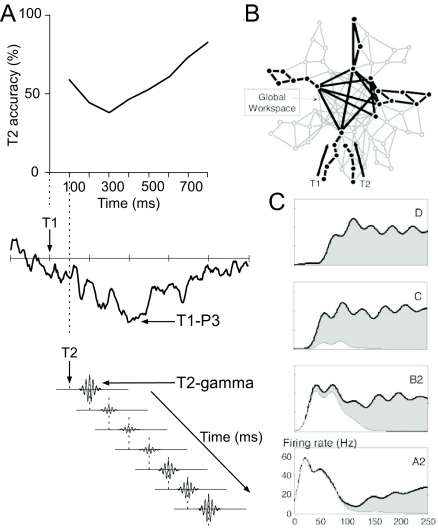
(a) Illustration of the model proposed by Fell, Klaver, Elger, and Fernandez (2002). According to this model the P3 that is evoked by the presentation of T1 and peaks around 400 ms after T1 impairs the early evoked gamma response for T2. The gamma response is assumed to be necessary for selection and identification of target stimuli and its impairment causes the attentional blink (AB). (b) Illustration of the application of the neuronal workspace model of conscious access to the AB as suggested by Dehaene, Sergent, and Changeux (2003). The schematic architecture of brain areas comprises multiple specialized processors and a central network of high-level areas temporarily interconnecting them. It is assumed that in the AB, T1 invades this “neuronal workspace” and areas lock into a single assembly supporting conscious reportability of T1. The invasion of the workspace by T1 blocks the processing of T2 at a similar depth thus causing the failure to report T2. (c) Neural activity evoked by seen and unseen T2 targets in recordings simulating the neuronal workspace model of conscious access. A2 and B2 refer to perceptual areas processing T2, C and D refer to higher association areas. In trials in which T2-related activity is evident in area D (T2 seen trials), simulated activity in lower areas C, B2, and A2 is characterized by long-lasting amplification (activity of area D and the resulting amplification are indicated in grey). If area D remains inactive, activity is short and mainly restricted to perceptual areas B2 and A2 (indicated in white). Sections (b) and (c) adapted from “A Neuronal Network Model Linking Subjective Reports and Objective Physiological Data During Conscious Perception” by S. Dehaene, C. Sergent, and J. P. Changeux, 2003), *Proceedings of the National Academy of Sciences of the United States of America, 100*(14), pp. 8521, 8524. Copyright 2003 by the National Academy of Sciences, U.S.A.

 To the best of our knowledge, the earliest neurocognitive theory on the AB suggested that suppression of evoked gamma oscillations may be the cause of the AB (Fell, Klaver, Elger, & Fernandez, 2002). Fell et al. (2002) reasoned that a process linked to T1 processing and indexed by the T1-related P3 impairs a T2 related process indexed by the early evoked gamma response to T2. The early evoked gamma response has been suggested to be necessary for attention allocation to a selected object and therefore for stimulus discrimination and target selection/identification (Debener, Herrmann, Kranczioch, Gembris, & Engel, 2003; Fell et al., 2002; Herrmann & Knight, 2001; Herrmann & Mecklinger, 2000, 2001; Sokolov et al., 1999; Tiitinen, May, & Näätänen, 1997; Tiitinen, Sinkkonen, May, & Näätänen, 1994). Based on the observations that (a) the AB reaches its peak at a T1-T2 interval of about 300 ms and that (b) the T1-related P3 had a peak latency of about 400 ms (McArthur, Budd, & Michie, 1999), the T2-related process was hypothesized to have a latency of about 100 ms. Because research failed to find impairments in ERPs occurring in this time period (Vogel et al., 1998), Fell et al. (2002) suggested that it is the early evoked gamma response that is impaired. The basic idea of the model is summarized in Figure 2a. 

 Dehaene, Sergent, and Changeux (2003) applied the global workspace model (Baars, 1998) to the AB. In this model it is proposed that conscious awareness of processed visual stimuli is related to the entry of the stimuli into a global brain state, which is described as a temporary state of connectivity between distant brain areas. It is hypothesized that during this period information becomes simultaneously available for multiple cognitive processes. The neural signatures of the global brain state would be long-lasting distributed activity and in particular gamma band emissions (Dehaene et al., 2003). With regard to the AB, Dehaene et al. (2003) suggest that both T1 and T2 go through an initial sensory processing stage by distinct neuronal assemblies (cf. Figure 2b and Figure 2c). Visual and semantic processing during this feed-forward sweep is assumed to be reflected in the P1, N1, and N400 ERPs, and, importantly, T1 and T2 do not inhibit one another at this stage. However, once T1 has entered the global workspace and has been subject to top-down amplification, T1 elicited inhibition will prevent T2 to enter the global workspace until the T1-related brain state has subsided. Because T2 fails to trigger long-lasting distributed activity and gamma band emissions during this period, T2 does not reach conscious awareness and no P3 waveform is generated. 

**Figure 3.**
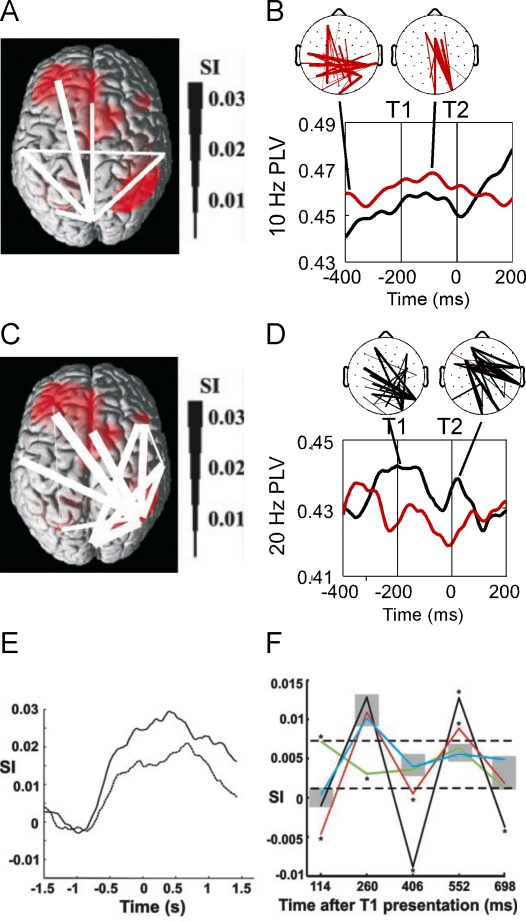
Stimulus (a, b) and target (c-f) related activity in the alpha and beta frequency bands. (a) Stimulus-related network identified by Gross, Schmitz, Schnitzler, Kessler, Shapiro, Hommel, and Schnitzler (2004). The network was found to primarily link occipital to left frontal areas. The degree of beta band synchronization of the stimulus-related connections was modulated at the stimulus presentation frequency (about 6.8 Hz). (b) Long-range synchronization as observed by Kranczioch, Debener, Maye, and Engel (2007). Synchronization at the stimulus presentation frequency of 10 Hz was increased for AB trials (red) as compared to no-AB trials (black), likely reflecting differences in distractor processing. As indicated by the topographic plots, differences in long-range synchronization were mainly due to higher synchronization between parieto-occipital and (left) frontal areas in AB trials. Note the similarity between the stimulus-related network in (a) and the pre-T1 activity of the distractor-related network in (b). (c)Target-related network identified by Gross et al. (2004). The strongest connections of the network were found between right posterior parietal regions and cingulum and left temporal and frontal regions. For target-related connections, synchronization in the beta band was modulated mainly by targets. (d) Long-range synchronization in the beta band as observed by Kranczioch et al. (2007). Synchronization was increased for no-AB (black) as compared to AB (red) trials, in particular between right temporo-parietal and left frontal and temporal electrode sites. Note the similarity between topographical patterns in (c) and (d). (e) Mean synchronization index (SI) for the target-related connections shown in (c). The no-AB condition (upper, solid line) is characterised by a stronger beta band synchronization than the AB condition (lower, dotted line). Conditions begin to differ clearly before T1 presentation. Zero corresponds to the presentation of T1. (f) Network synchronization to T1 and T2 (positive peaks at 260 and 552 ms after T1 presentation) and network de-synchronization to the distractors before and after the targets (negative peaks at 114, 406, and 698 ms). In AB trials (red line), both the T2-related synchronization and the distractor-related desynchronization are significantly attenuated. The black line corresponds to no-AB trials, the blue line to target related activation, and the green line to distractor related activity. Sections (a), (c), (e), and (f) adapted from “Modulation of Long-Range Neural Synchrony Reflects Temporal Limitations of Visual Attention in Humans” by J. Gross, F. Schmitz, I. Schnitzler, K. Kessler, K. Shapiro, B. Hommel, and A. Schnitzler, 2004, *Proceedings of the National Academy of Sciences of the United States of America, 101*(35), pp. 13052, 13053. Copyright 2004 by the National Academy of Sciences, U.S.A. Sections (b) and (d) adapted from “Temporal dynamics of access to consciousness in the attentional blink” by C. Kranczioch, S. Debener, A. Maye, and A. Engel, 2007), *NeuroImage*, 37(3), p. 953.

 Both models have in common that they assume that a process related to T1 processing inhibits a T2-related process that is reflected in gamma band oscillations. Fell et al. (2002) propose that the impaired process is reflected in the early evoked gamma band response. Though not explicitly stated, the proposal by Dehaene et al. (2003) is more compatible with the assumption that the induced gamma band response is impaired (cf. Figure 3e; Dehaene et al., 2003). Induced gamma band responses have, among others, been linked to conscious perception (Engel, Fries, König, Brecht, & Singer, 1999; Schurger, Cowey, & Tallon-Baudry, 2006; Summerfield, Jack, & Burgess, 2002; but see also Schurger, Cowey, Cohen, Treisman, & Tallon-Baudry, 2008). As will be discussed below, direct empirical evidence for or against either model is still very sparse. 

## Empirical findings on oscillatory activity in the attentional blink

### Theta

 Theta oscillations have been related to meditation (Cahn & Polich, 2006) and to memory function (Jensen & Tesche, 2002; Sauseng, Griesmayr, Freunberger, & Klimesch, 2010). Slagter et al. (2009) compared AB performance and brain activity in novices and practitioners in meditation before and after a 3-month period of intense meditation training. On the behavioural side, they found that the AB was significantly reduced in the practitioner group after the meditation training. T1 performance was significantly better after the meditation training in both groups. After detected T1-targets as well as after detected T2-targets theta inter-trial phase consistency was enhanced. The post-T2 theta inter-trial phase consistency to successfully identified T2 targets was affected by meditation training in that it increased over right ventro-lateral and midline frontal regions. This effect was restricted to the meditation practitioners as was the reduction of the behavioural AB deficit after meditation training. 

With regard to the processing of T2, the results of this study indicate that it benefits from an increase in theta synchronization after its presentation. Furthermore meditation training appears to have a bene-ficial effect on theta synchronization and performance in the AB task (Slagter et al., 2009). Frontal theta oscillations have been found to be related to the amount of information that needs to be held in working memory during task performance (Jensen & Tesche, 2002). Recently interregional theta synchronization has been suggested to play a role in the integration of various brain areas and therefore be important for working memory control processes (Sauseng et al., 2010). In view of previous research the post T1 and T2 increase in theta phase locking could represent a working memory process necessary for updating and/or integrating information into working memory.

### Alpha

Alpha oscillations have been mainly related to cognitive processing in the field of memory, visual attention, and perception (Herrmann et al., 2002). More specifically, an increase in alpha activity prior to stimulus presentation and a decrease in alpha activity during stimulus processing have been linked to improved memory task performance (Klimesch, 1999; Klimesch, Sauseng, & Hanslmayr, 2007). Perceptual performance however seems to benefit from the opposite pattern: Here a decrease in alpha activity prior to stimulus presentation has been related to better target detection (Hanslmayr et al., 2005; Klimesch et al., 2007; van Dijk, Schoffelen, Oostenveld, & Jensen, 2008), likely reflecting the successful direction and deployment of attention (Hanslmayr et al., 2007; Thut, Nietzel, Brandt, & Pascual-Leone, 2006). The link between alpha amplitude and perception has recently been shown to be of causal and not just correlational nature (Romei, Gross, & Thut, 2010). Moreover, the phase of alpha activity seems to play a significant role in modulating the visual detection threshold (Busch, Dubois, & VanRullen, 2009; Mathewson, Fabiani, Gratton, Beck, & Lleras, 2010; Mathewson, Gratton, Fabiani, Beck, & Ro, 2009; Romei et al., 2010). Both attention and memory function have been discussed as contri-buting to the AB and thus a relation between alpha activity and the AB can be expected.

This prediction however is somewhat complicated by the rapid serial visual presentation (RSVP) method employed in most AB studies. Oscillations in the visual cortex are known to synchronize to the pre-sentation frequency of visual stimuli, resulting in the ssVEP (Müller, Teder, & Hillyard, 1997; Regan, 1966). Using the RSVP method with a rate of 10 stimuli per second (which is typical for the AB) will therefore evoke an ssVEP brain response at 10 Hz, and likely harmonic and/or subharmonic responses (Herrmann, 2001; Vialatte et al., 2010). It is evident that this response could obscure modulations of, or interact with, the intrinsically generated alpha responses. On the other hand, an interesting property of the ssVEP is that its amplitude increases when the stimulus stream is attended compared to when it is not actively attended (Müller & Hillyard, 2000; Müller & Hübner, 2002; Müller, Malinowski, Gruber, & Hillyard, 2003). This feature could make it even harder to tear apart whether an effect in the alpha frequency band reflects changes in intrinsically generated alpha or ssVEP activity.

 Keil and colleagues (2006) used an AB paradigm with stimuli varying in emotional content (pleasant, neutral, unpleasant) presented at a frequency of 8.6 Hz. They found a post-stimulus increase in amplitude at the stimulation frequency for arousing T2 stimuli as compared to neutral T2 stimuli starting approximately 120 ms after T2 onset. Furthermore, irrespective of emotional content, for T1 this target-related response was found to be significantly reduced in no-AB as compared to AB trials, whereas it was increased for T2 in no-AB as compared to AB trials. The performance related modulation of the T1-ssVEP response was successfully replicated in a later study (Keil & Heim, 2009). 

 Kranczioch et al. (2007) compared no-AB and AB trials in a standard AB paradigm and found significantly smaller inter-area phase locking for the no-AB versus AB trials starting before T1 presentation until after T2 presentation. In line with previous findings (Gross et al., 2004), increased inter-area phase locking was most evident between occipito-parietal and left frontal electrode sites (cf. Figure 3a and Figure 3b). The difference in inter-area phase locking was most pronounced at the stimulus presentation frequency of 10 Hz. Amplitude and inter-trial phase consistency where also significantly smaller in this time range for no-AB trials, thought the effect was not as sustained and only significant in a time window before T1 presentation. Furthermore, no-AB trials were associated with an inter-area phase locking increase at 13 Hz just before T1 presentation until just after T2 presentation. The increase in inter-area phase locking was widespread but excluded fronto-central electrode sites and thus was clearly different from the 10 Hz effect.

 MacLean and Arnell (2011) analysed alpha power between 10 and 12 Hz in a 2 s period prior to the onset of the RSVP sequence. The expectation of the onset of the RSVP sequence reduced alpha activity in general. This effect was most pronounced at right frontal electrode sites. This reduction in pre-RSVP alpha activity was stronger in AB-trials as compared to no-AB trials, but only if T2 was presented inside the AB window. If T2 was presented outside the AB window the opposite pattern was observed, that is, now AB trials were associated with a smaller reduction in pre-RSVP alpha power than no-AB trials. 

 In their study on the effect of meditation on the AB and oscillatory brain activity discussed above, Slagter and colleagues (2009) observed that in addition to the post-target theta effects oscillatory alpha activity was related to the AB. In detail, they found after meditation practice occipital alpha inter-trial phase consistency to be reduced for meditation practitioners in the no-AB trials several hundred milliseconds prior to T1. In addition, practitioners showed an increase in the amplitude of the T1-induced alpha response in no-AB trials as compared to novices after 3 months of meditation training. 

 Finally, a study by Martens et al. (2006) observed differences in distractor-related activity, that is, the ssVEP, between individuals who do not have an AB (non-blinkers) and those who do (blinkers). SsVEP amplitude was found to be significantly enhanced for blinkers compared to non-blinkers for the whole RSVP-period. In line with this finding, Wierda, van Rijn, Taatgen, and Martens (2010) found that when AB performance is improved by introducing a concurrent task, ssVEP amplitude is reduced in the concurrent task condition. 

 The picture emerging from this research is one in which an increase in activity in the alpha band before the appearance of T1 and thus during the presentation of the distractor stream is detrimental to AB performance (Kranczioch et al., 2007; Martens et al., 2006; Slagter et al., 2009; Wierda et al., 2010). It seems likely that this alpha effect is largely the result of a modulation of the RSVP-related ssVEP. As the ssVEP has been shown to increase if attention is directed to the stimulus stream (Müller & Hillyard, 2000; Müller & Hübner, 2002; Müller et al., 2003), this alpha band modulation could reflect the overinvestment of processing resources to the distractors and, potentially, to T1 (Olivers & Nieuwenhuis, 2006). The finding of MacLean and Arnell (2011) that alpha power before the onset of the RSVP, where no ssVEP is present, is actually reduced in AB as compared to no-AB trials is in support of the (over-)investment theory, as a reduction of intrinsically generated alpha activity has been linked to anticipatory attentional investment (Onoda et al., 2007; Yamagishi, Goda, Callan, Anderson, & Kawato, 2005). The differences in intrinsically generated alpha activity observed by MacLean and Arnell (2011) are seconds away from the presentation of both T1 and T2. However, some findings also link an increase in alpha activity around T1-presentation to escaping the AB (Kranczioch et al., 2007; Slagter et al., 2009). At least for one of the studies (Kranczioch et al., 2007), this effect was strongest outside the ssVEP frequency range. Since alpha activation has been linked to active functional inhibition (Klimesch, Doppelmayr, Schwaiger, Auinger, & Winkler, 1999; Klimesch et al., 2007; van Dijk et al., 2008), this increase in alpha activity could reflect the (partial) inhibition of T1 processing that is required to free resources for successful T2 processing in the attention-demanding AB task. The results of Keil and colleagues (2006, 2009) support this general idea and provide further evidence that the amount of resources invested into stimulus processing is reflected in the ssVEP. A challenge for future studies will be firstly to disentangle the effects of spontaneous alpha and of the ssVEP in the AB and se-condly to study the interrelation between the two signals. Research is currently under way in our lab that aims to answer these important questions.^1^

### Beta

 Beta activity has classically been linked to sensorimotor processing. It typically is suppressed in the primary sensorimotor region of the active body part during motor action but shows an increase just after (Herrmann et al., 2002). Despite this strong connection to sensorimotor processing, beta oscillations are also frequently found in non-motor tasks (Buschman & Miller, 2007, 2009; Gross et al., 2004, 2006; Kranczioch et al., 2007). Interestingly, one of the first studies to identify a role for beta-oscillations in a cognitive task was an AB study (Gross et al., 2004). In an attempt to unify the wide range of findings on beta band activity Engel and Fries (2010) recently suggested that beta oscillations indicate the tendency of a system to maintain a status quo or cognitive state, especially in tasks requiring endogenously driven top-down control. 

 The study by Gross et al. (2004) used the magneto-encephalogram (MEG) to study oscillatory activity in the AB. Beta inter-area phase locking was found to be generally increased in a target-related network consisting of frontal, temporal, and parietal areas for no-AB trials compared to AB trials (Figure 3c). Interestingly, the increase started considerably before the presentation of T1 (Figure 3e). Moreover, beta inter-area phase locking of the target-related network was found to be modulated as a function of performance. A significant phase synchronization peak occurred around 260 ms after both T1 and T2 presentation, the T2-related peak was significantly attenuated in AB trials. In addition, for distractor stimuli that preceded and followed T1 and T2 a strong desynchronization of the network was observed, which was again attenuated in AB trials (Figure 3f). A later re-analysis of the original data indicated that the post-T1 desynchronization-synchronization pattern becomes more pronounced with increased T1 probability (Gross et al., 2006). 

 In line with these results are the findings by Kranczioch et al. (2007) , reporting an increase in beta inter-area phase locking for no-AB trials just before T1 presentation until just after T2 presentation. This increase in inter-area phase locking took primarily place over right temporo-parietal and left frontal regions (Figure 3d), nicely paralleling the pattern of inter-area phase locking found by Gross and colleagues (2004; cf. Figure 3c). 

 From both the research of Gross and colleagues (2004, 2006) and Kranczioch and colleagues (2007) it appears that beta inter-area phase locking in a fronto-temporo-parietal network is beneficial for T2 detection. Synchronization differences are evident even before the presentation of T1 and continue throughout the trial. This fits well with the idea of beta oscillations being related to maintaining the cognitive set or status quo in tasks requiring endogenously driven top-down control (Engel & Fries, 2010). Yet of particular relevance for task performance in the AB seems to be a rapid switch between synchronization of the network in response to targets and desynchronization in response to distractors. This beta synchronization/desynchronization could be a mechanism that enhances target processing and at the same time avoids interference from distractors (Gross et al., 2004,^2006^). A somewhat different though not unrelated interpretation of the data is that in particular the desynchronization between T1 and T2 could be an essential mechanism for allowing the transition between two stable oscillatory states: Only if the T1-related stable state is sufficiently desynchronized T2 can enter its stable (synchronized) state and be reported. Suppression of distractors would be a by-product of the stable states and the destabilisation between them (Gross et al., 2004; Kessler, Gross, Schmitz, & Schnitzler, 2006; Kessler et al., 2005). The idea that desynchronization is required for the transition between two stable states was originally proposed by Rodriguez and colleagues (Rodriguez et al., 1999). In the context of the AB, it is compatible with the delayed attentional reengagement account by Nieuwenstein and colleagues discussed above (Nieuwenstein, 2006; Nieuwenstein & Potter, 2006; Nieuwenstein et al., 2005, 2009) as well as with the basic idea (though not the proposed frequency) of the global workspace account (Dehaene et al., 2003). 

### Gamma

Gamma oscillations have been related to a wide variety of cognitive processes such as memory, attention, or learning (Engel, Fries, & Singer, 2001; Herrmann, Fründ, & Lenz, 2010; Herrmann & Kaiser, 2010; Herrmann, Munk, & Engel, 2004; Tallon-Baudry, 2009). Early evoked gamma band responses are generated in early sensory cortices but are nevertheless under the influence of top-down processes such as attention and memory. Induced gamma band responses seem to represent later processing stages and can thus be observed in many different brain areas (Herrmann & Kaiser, 2010).The synchronization of gamma activity between brain areas seems to play an important role for integrating distributed neuronal processes (Fries, 2009; Varela et al., 2001).^2^

 Kranczioch (2004) investigated the proposal that the early evoked gamma band response is impaired in the AB (Fell et al., 2002). Even though T1 and T2 evoked a P3 ERP in this study, no evoked gamma band response was observed to either target and hence the early evoked gamma account of the AB (Fell et al., 2002) could not be tested. Kranczioch (2004) suggested that the failure to observe an early evoked gamma response could be due to the temporal coincidence of the response and the presentation of the targets’ masks, the size of the stimuli, as small stimuli hardly evoke an early gamma response (Busch, Debener, Kranczioch, Engel, & Herrmann, 2004), or that the number of trials entering analysis was not large enough to raise the signal-to-noise ratio sufficiently. These ideas were examined in a subsequent study (Kranczioch, 2004; Kranczioch, Debener, Herrmann, & Engel, 2006). In order to increase the number of events that could enter analysis, this study did not apply the AB paradigm. Participants observed a continuous RSVP stream that contained target items that were at least 1.5 s apart. To test whether the temporal coincidence of the expected early evoked gamma response to the target and the occurrence of the item that followed the target was a critical, RSVP presentation frequencies of 10 and 7.1 Hz were compared. Moreover, the size of the stimuli was varied. Again no early evoked gamma response was observed. The authors suggested that this could be due to the high-amplitude ssVEP produced by RSVP which could effectively mask the low-amplitude early evoked gamma band response in the scalp-recorded EEG (Kranczioch et al., 2006). If this were indeed the case it would pose a serious problem for testing the early evoked gamma account of the AB (Fell et al., 2002), as one major contribution to the AB is the RSVP stream (but see, e.g., Visser, Bischof, & Di Lollo, 2004). Interestingly, Kranczioch et al. (2006) did observe a late induced gamma band response that was more pronounced at a stimulus presentation frequency typical for the AB, that is, 10 Hz, as compared to 7.1 Hz. This response was not affected by stimulus size and was, in accordance with the “match-and-utilization-model” of gamma band responses proposed by Herrmann and colleagues (2004) , argued to reflect a temporal signature of neural interactions leading to updating of working memory. 

 Nakatani and colleagues (2005) studied the role of inter-area phase locking in the AB. Inter-area phase locking was found to be enhanced across the whole head in the experimental condition where both T1 and T2 had to be detected, as compared to the control condition where only T2 was task relevant. Just before the presentation of T1 inter-area phase locking was enhanced for no-AB trials, but only if T2 followed T1 closely. A similar anticipatory enhancement was not observed in AB trials. If T2 was presented outside the AB time window inter-area phase locking before the presentation of T1 was generally low and did not differentiate between AB and no-AB trials (see Figure 4 of Nakatani et al., 2005). Accordingly, Nakatani et al. (2005) argued that the enhancement in anticipatory gamma inter-area phase locking reflects baseline attention and the recruitment of processing capacity. They further suggested that these processes play a less important role if T2 is presented outside the AB time window. 

**Figure 4.**
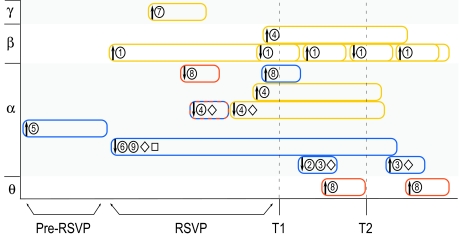
Summary of the empirical findings of studies on oscillatory brain activity in the attentional blink. Findings are sorted by frequency bands (theta and beta highlighted in white, alpha and gamma highlighted in grey) and are shown in approximate temporal relation to T1 and T2 presentation. Yellow indicates changes in inter-area phase locking, blue codes for amplitude changes, and red codes for inter-trial phase consistency changes. Arrows indicate the direction of the change. Upward arrows indicate that an increase of activity has been linked to good performance in the AB task, whereas downward arrows indicate that a decrease in activity has been linked to good performance in the AB task. The numbers indicate the corresponding study. The diamond indicates findings that likely reflect the steady-state visual evoked potential (ssVEP). The square indicates studies that only looked at activation related to distractors. (1) Gross, Schmitz, Schnitzler, Kessler, Shapiro, Hommel, and Schnitzler (2004) and Gross, Schmitz, Schnitzler, Kessler, Shapiro, Hommel, and Schnitzler (2006). (2) Keil and Heim (2009). (3) Keil, Ihssen, and Heim (2006). (4) Kranczioch, Debener, Maye, and Engel (2007). (5) MacLean and Arnell (2011). (6) Martens, Munneke, Smid, and Johnson (2006). (7) Nakatani et al. (2005). (8) Slagter, Lutz, Greischar, Nieuwenhuis, and Davidson (2009). (9) Wierda, van Rijn, Taatgen, and Martens (2010).

 Despite the many reports on gamma band activity in a wide variety of cognitive domains, from the research summarized above no clear picture emerges for the role of gamma band activity in the AB. The studies by Kranczioch (2004) and Kranczioch et al. (2006) indicate that it might be particularly difficult to observe early evoked gamma band responses in a typical AB paradigm, making it in turn difficult to test any hypothesis regarding the functional role of these responses as for instance proposed in the model by Fell and colleagues (2002). The baseline attention interpretation provided by Nakatani et al. (2005) to account for their findings seems reasonable. However, the question remains why anticipatory synchronization should generally be smaller for long T1-T2 lags. That is, if short-lag and long-lag trials are presented randomly as in the study by Nakatani and colleagues (2005) , on average anticipatory synchrony should be comparable for short-lag and long-lag trials, even though it might make a difference only for short-lag trials. Models on the role of oscillatory activity in the AB propose that the inhibition of post-T2 gamma activity may cause the AB (Dehaene et al., 2003; Fell et al., 2002). The empirical studies reviewed above have so far failed to provide evidence in support of these models. 

## Emerging picture from empirical studies

The picture emerging from the research on oscillatory activity reviewed here shows that the successful identification of both targets in an AB paradigm relates to a dynamic interplay of oscillations at different frequencies occurring at different moments in time. As is illustrated in Figure 4, even before the first target is shown, AB and no-AB trials differ systematically. Pre-T1 inter-area phase locking has been suggested to be beneficial to T2 performance in the AB task (Gross et al., 2004; Kranczioch et al., 2007; Nakatani et al., 2005). Moreover, relatively higher alpha power in expectation of an RSVP trial, reduced distractor-related activity and decreases in both power and synchrony in the alpha frequency range just before T1 onset have been linked to escaping the AB (Kranczioch et al., 2007; Martens et al., 2006; Slagter et al., 2009; Wierda et al., 2010). Differences in oscillatory activity continue after the presentation of the first target. Around T1 an increase in alpha power becomes apparent in no-AB trials (Slagter et al., 2009) that lasts until after T2 presentation (Kranczioch et al., 2007). The ssVEP response to T1 is reduced in no-AB trials while at the same time the T2-ssVEP response is enhanced (Keil & Heim, 2009; Keil et al., 2006; Kranczioch et al., 2007; Martens et al., 2006; Wierda et al., 2010). Successful target detection is furthermore linked to target-related synchronization increases after T1 and T2 in the theta and beta bands (Gross et al., 2004, 2006; Kranczioch et al., 2007; Slagter et al., 2009) and systematic desynchronization in the beta band (Gross et al., 2004, 2006).

### Pre-T1 activity

 Differences in pre-T1 alpha activity (Kranczioch et al., 2007; Slagter et al., 2009) during the RSVP stream most likely reflect differences in the ssVEP. The reduction of this activity in no-AB trials may represent a restriction of resources devoted to the processing of T1 and/or the distractor stream, an interpretation supported by the observed performance-related modulations of the distractor ssVEP (Martens & Valchev, 2009; Wierda et al., 2010) as well as the performance-related modulations in alpha activity in expectation of the RSVP stream (MacLean & Arnell, 2011). Pre-T1 or anticipatory beta inter-area phase locking (Gross et al., 2004, 2006; Kranczioch et al., 2007) could reflect preparation of the system, which would allow faster succession of stable states. This is generally in line with the interpretation of anticipatory gamma band inter-area phase locking reflecting the recruitment of processing resources put forward by Nakatani et al. (2005) . It might also reflect a top-down process responsible for the retention of processing resources. This fits well with the suggestion that beta activity is related to endogenously driven top-down attention which helps to maintain the current cognitive set and gives it priority over new signals (Engel & Fries, 2010). According to this idea one would not expect an anticipatory increase in gamma activity in no-AB trials though, as this would facilitate the conveyance of bottom-up signals (Buschman & Miller, 2007; Engel & Fries, 2010). This is in conflict with the findings of Nakatani et al. (2005) , and more research is clearly needed in order to solve this contradiction. 

The studies on oscillatory activity in the AB provide converging evidence that whether in a given instance an AB occurs or not is related to the pre-T1 state of the brain. This opens a new perspective on the mechanisms underlying the AB that neither ERP nor fMRI research could so far provide, but is in line with the general notion that the current state of the brain modulates stimulus evoked responses and the processing of incoming information (Arieli, Sterkin, Grinvald, & Aertsen, 1996; Fontanini & Katz, 2008). Whether creating a brain state that is particularly advantageous for the task demands of the AB is under volitional control and whether the pre-T1 brain state is causally linked to the occurrence of the AB remains to be studied and is one of the main interests of our work.

### Post-T1 activity

Differences in oscillatory activity continue after the presentation of the first target and are evident in the alpha band (Kranczioch et al., 2007; Slagter et al., 2009), the ssVEP (Keil & Heim, 2009; Keil et al., 2006), the theta (Slagter et al., 2009), and beta bands (Gross et al., 2004, 2006; Kranczioch et al., 2007). In detail, improved performance in the AB has been linked to increased alpha activity around and after the presentation of T1, which could indicate the activity of inhibitory processes (Klimesch et al., 2007). Inhibition could help to prevent that T1 and the surrounding distractors receive too much of a capacity-limited resource. This fits well with the result that no-AB trials are associated with a relatively smaller T1-ssVEP response and a relatively larger T2-ssVEP response, respectively thought to reflect inhibition and facilitation of early sensory processing (Keil & Heim, 2009; Keil et al., 2006). It is interesting to note that in line with ERP studies (Dell’Acqua et al., 2006; Jolicoeur et al., 2006; Kranczioch et al., 2007; Sergent et al., 2005; Vogel et al., 1998) oscillation studies did not observe differences between target-evoked activity in AB and no-AB trials before this ssVEP response, that is, at about 170-200 ms. Thus, oscillation data so far support the view that with regard to the processing of target stimuli the AB operates at a stage after initial sensory processing even though early, pre-target anticipatory synchronization may set the stage for the differences in processing.

Analyses of beta band synchronization patterns in the time period of T1 and T2 presentation indicated that successful target processing is associated with target-related synchronization and, in interestingly, systematic desynchronization (Gross et al., 2004, 2006). This synchronization/desynchronization pattern could reflect a mechanism for enhancing target processing and suppressing the processing of distractors within a network consisting of areas relevant for target detection, visual attention, and working memory that is particularly relevant for avoiding the AB (Gross et al., 2004). However, in particular the desynchronization between the T1-related synchronization and the T2-related synchronization could also reflect an essential mechanism required for the successful transition between two stable oscillatory states. In this scenario, suppression of distractors would be a by-product of the stable states and the destabilisation between them (Gross et al., 2004; Kessler et al., 2005, 2006). The enhancement of theta synchronization after T1 and T2 for successfully indentified targets fits very well in this picture and may in particular reflect the working memory component of this network (Slagter et al., 2009).

Once T1 is presented, the timing and interplay of facilitatory and inhibitory or modulating processes in the form of beta and theta oscillations seems to be of particular importance. Escaping the AB is most likely if the processing of distractors close to T1 and T2 can be suppressed and if the resources that are directed at T1 and that facilitate its processing are somewhat reduced, in favour of T2. Whether in a given trial this interplay can operate successfully could depend on the timely and sufficient conveyance of top-down signals. Top-down processing has been linked to beta band activity (Engel & Fries, 2010). Insufficient top-down control could lead to deficits in both the inhibition of distractors and the attenuation of T1 processing or to a slower succession of stable states and a lack of facilitation of T2 processing. If this is correct then beta band activity should not only be reduced for AB as compared to no-AB trials as has been shown previously (Gross et al., 2004; Kranczioch et al., 2007) but could also be expected to be reduced or delayed for so-called blinkers that show a large AB deficit (Feinstein et al., 2004; Martens et al., 2006; Martens & Valchev, 2009). A similar prediction can be made for standard AB setups as compared to setups that have been found to reduce the AB (Olivers & Nieuwenhuis, 2005, 2006; Taatgen, Juvina, Schipper, Borst, & Martens, 2009; Wierda et al., 2010); again a larger AB deficit should be linked to reduced beta band activity. However, the finding that AB performance can improve without an accompanying change in beta activity (Slagter et al., 2009) indicates that modulations of beta activity are only part of the story.

## Conclusion

 In a recent extensive review of AB theories and behavioural data, Dux and Marois (2009) argue that none of the AB models can account for all the findings in the literature and that therefore the most likely scenario is that the AB has a multifactorial origin. They leave however open the possibility that these multiple processes rely on a common capacity-limited resource, which, however, would again fall short to explain all the findings. Along similar lines, Hommel and co-workers (2006) conclude from the neuroscientific evidence that it is unlikely that the AB can be tracked down to a single cortical structure or system, but that it seems that the AB arises from the fact that several components have to interact as a network. The problem is that communication within this network can refer to only one topic at a time, effectively creating a bottleneck for target processing. The empirical evidence Hommel and colleagues (2006) could draw upon at that time suggested that the communication within the network and in particular the bottleneck are tightly linked to beta band synchronization and desynchronization during target processing. Research on oscillatory brain activity in the AB published since then adds to this that task-relevant communication within the network may also be evident in other frequency bands at varying latencies, and that a modulation in the AB can occur without an accompanying modulation in beta activity. Taking a closer look at these recent findings and their interactions with beta band activity and performance and introducing experimental manipulations of oscillatory brain activity will not only help to better understand the AB, but also why the mechanism creating the AB, whatever its nature, can still be bypassed in conditions that should normally result in blinking the target. 

## Footnote

1 See Hanslmayr, Gross, Klimesch, and Shapiro (2011) for a detailed discussion of the potential role and interplay of alpha amplitude, phase, and inter-area phase locking.

2 It should be noted that recent studies by Yuval-Greenberg and colleagues (Yuval-Greenberg & Deouell, 2009; Yuval-Greenberg, Tomer, Keren, Nelken, & Deouell, 2008) suggest that many findings of induced gamma band responses, and in particular in EEG data, might not reflect neural activity but are the result of minute movements of extraocular muscles, called microsaccades (for a recent review of this topic, see Schwartzman & Kranczioch, 2011).
